# Large-scale synthesis of free-standing N-doped graphene using microwave plasma

**DOI:** 10.1038/s41598-018-30870-3

**Published:** 2018-08-22

**Authors:** N. Bundaleska, J. Henriques, M. Abrashev, A. M. Botelho do Rego, A. M. Ferraria, A. Almeida, F. M. Dias, E. Valcheva, B. Arnaudov, K. K. Upadhyay, M. F. Montemor, E. Tatarova

**Affiliations:** 1 Instituto de Plasmas e Fusão Nuclear, Instituto Superior Técnico, Universidade de Lisboa, Lisboa, 1049 Portugal; 2 Faculty of Physics, Sofia University, 1164 Sofia, Bulgaria; 3 CQFM-Centro de Química-Física Molecular and IN and IBB-Institute for Bioengineering and Biosciences, Instituto Superior Técnico, Universidade de Lisboa, 1049-001 Lisboa, Portugal; 4 Centre of Physics and Engineering of Advanced Materiais, Instituto Superior Técnico, Universidade de Lisboa, 1049-001 Lisboa, Portugal; 5 Centro de Química Estrutural (CQE), Departamento de Engenharia Química, Instituto Superior Técnico, Universidade de Lisboa, Lisboa, 1049 Portugal

## Abstract

Direct assembling of N-graphene, i.e. nitrogen doped graphene, in a controllable manner was achieved using microwave plasmas at atmospheric pressure conditions. The synthesis is accomplished via a single step using ethanol and ammonia as carbon and nitrogen precursors. Tailoring of the high-energy density plasma environment results in a selective synthesis of N-graphene (~0.4% doping level) in a narrow range of externally controlled operational conditions, i.e. precursor and background gas fluxes, plasma reactor design and microwave power. Applying infrared (IR) and ultraviolet (UV) irradiation to the flow of free-standing sheets in the post-plasma zone carries out changes in the percentage of sp^2^, the N doping type and the oxygen functionalities. X-ray photoelectron spectroscopy (XPS) revealed the relative extension of the graphene sheets π-system and the type of nitrogen chemical functions present in the lattice structure. Scanning Electron microscopy (SEM), Transmission Electron microscopy (TEM) and Raman spectroscopy were applied to determine morphological and structural characteristics of the sheets. Optical emission and FT-IR spectroscopy were applied for characterization of the high-energy density plasma environment and outlet gas stream. Electrochemical measurements were also performed to elucidate the electrochemical behavior of NG for supercapacitor applications.

## Introduction

Beyond its unique set of physico-chemical properties, graphene can be considered as a robust, atomic scale scaffold from which other 2D materials can be derived through the attachment of foreign atoms and functional groups^1–4^. Among the possible doping agents, nitrogen has drawn a considerable amount of attention because its atomic radius is comparable to that of carbon and contains five valence electrons available to form strong covalent bonds. The conjugation between the nitrogen lone-pair electrons and the graphene π-system modifies graphene physical and chemical properties. The substitution of carbon atoms by nitrogen ones influences the atomic charge distribution on the graphene scaffold and creates “active sites” thus significantly increasing the electrochemical activity of nitrogen-doped graphene, known as N-graphene (NG)^4^.

The theoretical studies^4,5^ predicted modified electronic and chemical properties of nitrogen-doped graphene, which is being supported by numerous experimental investigations. Indeed, there is significant experimental evidence of the advanced properties of the NG-based materials in respect to already practically implemented ones^4,5^. For instance, NG has been tested in fuel cells either as the catalyst or carbon supports. The “active sites” can be engaged in catalytic reactions, such as oxygen reduction reaction (ORR), or can be used as a scaffold for other catalysts^3–8^. NG demonstrates better chemical reactivity and sheet-to-sheet separation than the pristine graphene^9^. To this end, N-doping is a very promising approach for the development of metal-free carbon-based catalysts with even better performance than commercially available Pt-based electrodes, with prospective impact on fuel cells’ commercialisation. However, the lack of an appropriate synthesis route providing both high quality and quantity of NG hinders its practical implementation.

Graphitic, pyridinic and pyrrolic are the three common N-bonding configurations. Graphitic N can facilitate electron transfer by lowering charge-transfer resistance of the electrode. Being electrochemically active, pyridinic and pyrrolic N offer high pseudocapacitance^5^. The influence of the N-bonding configuration on the carrier concentration and consequently on the electronic properties of graphene was studied in^10^.

N-doped graphene display interesting properties for charge storage applications. Supercapacitors with high capacitance (~280 Fg^−1^), excellent life number of cycles (>200,000), suitable to operate with flexible substrates and various electrolytes were developed in^11^ using nitrogen-doped graphene. Moreover, N-doped graphene has been hybridized with different metal compounds to enhance electronic and ionic conductivity^12^.

N-doped graphene nanoribbons were also tested as conductive host materials in lithium-sulfur (Li-S) batteries, demonstrating stronger fixation of sulfur-containing species and higher stability, as compared with pristine graphene nanoribbons^13^. Applications of N-graphene in direct electrochemistry of glucose oxidase and glucose biosensing were studied^14^. Moreover, the emerging N-graphene applications include also areas such as gas storage, sensors, light processing (LEDs), spintronics, etc.^15–26^. The sensing ability of NG was investigated for certain dyes by a new surface analysis technique, i.e. graphene-enhanced Raman scattering^15^. It was demonstrated that NG can be used to efficiently detect trace amounts of certain molecules.

Rechargeable zinc-air battery with the nitrogen-doped graphene nanoribbons used as an air electrode in a two-electrode configuration was assembled in^27^. The battery exhibited an open-circuit voltage of 1.46 V, a specific capacity of 873 mAhg^−1^, and a peak power density of 65 mWcm^−2^, that can be continuously charged-discharged with an excellent cycling stability.

Numerous methods for synthesis of N-graphene^4,5^ can be categorized into *in-situ* direct methods and post-treatment approaches. *In situ* approaches characterized by simultaneous graphene synthesis and N-doping include CVD, ball milling, solvothermal synthesis, etc^4,5,18,19^ CVD involves growth of N-graphene on a preheated structure (typically nickel or copper) in the carbon and nitrogen-containing environment. Although CVD is one of the most common methods for *in-situ* NG synthesis, it suffers from metal interference. Post-treatment methods include wet chemical methods, thermal annealing of graphene oxides (GO) with heteroatom precursors and plasma-based treatments^28–38^. These methods present several drawbacks, such as degradation of the nanostructure properties due to the transition metals used, the requirement of expensive and/or hazardous catalysts, vacuum systems and very high temperatures, and lengthy/complex batch procedures. Although, in principle, direct synthesis may have the potential to create homogeneous doping throughout the bulk material, the results reported up to now fail to indicate so. As a rule, graphene oxide (GO), in the presence of N-precursors, has been frequently used for N-graphene synthesis. However, chemical reduction of GO requires the use of reducing toxic agents. Although it is a low-cost method to produce N-graphene in a relatively large scale, the materials obtained exhibit only relatively low electrical conductivity due to the contaminants, saturated sp^3^ bonds and bonded oxygen groups^28–30^. Thus, restoration of the high conductivity of N-graphene sheets is an issue.

Graphene/N-graphene´s properties vary strongly as a function of its synthesis method and their intrinsic properties are drastically dependent on the structural quality. A good spatial control of the energy and mater fluxes at a nanoscale, is indispensable in enabling a cost-effective and environmentally friendly synthesis process and constitutes one of the main challenges of the above-mentioned conventional methods. Existing commercial products (e.g.: https://graphene-supermarket.com/) show variable physical properties, thus limiting their use. Generally, the fabrication of N-graphene meets significant unresolved problems such as: i) uncontrolled N bonding type and distribution; ii) N doping at non-specific positions and untuned doping content. Most importantly, the greatest challenge on the path towards either graphene or N-graphene commercialization is the controllable production of high quality material on a large scale, at low cost and in a reproducible manner. To this end, plasmas and their ability to control energy and matter at an atomic scale level appear as powerful tools to tackle this challenge^2,3^. Plasma assisted growth of nanostructures can be achieved without the use of catalysts due to plasma’s unique ability to activate the surface, thus creating favorable conditions for nucleation and growth processes.

The successful synthesis of N-graphene consisting of multiple layers with 1% level of doping using a DC arc-discharge with carbon electrodes in an atmosphere of ammonia, was reported in^34^. The majority of plasma-induced N-doping methods, however, employ the post-treatment approach, commonly treating graphene with N_2_ or ammonia plasma^31–33^. A doping level in the range 5–15% at the surface of graphene and Highly Oriented Pyrolytic Graphite (HOPG) was achieved using low pressure RF nitrogen plasma treatment^39^. Similarly, microwave N_2_-Ar post discharge plasma was used for N-doping of free-standing graphene, with a maximum level of doping achieved of 5.6%^32^. Gas-phase controllable doping of graphene by ammonia plasma exposure, with maximum doping level of about 0.4% has been reported in^32^. However, the large percentage of oxygen, common for post-treatment approaches, is to be noticed. Generally, plasma-based post-treatment approaches can provide higher levels of N-doping compared to direct ones, but these processes have some disadvantages. They are two-step processes, slower, more complex and expensive, and are commonly performed at low-pressure conditions. Furthermore, plasma post-treatment affects only the surface of the sample, which significantly reduces its effectiveness.

In the present work, *in-situ* direct synthesis of free-standing N-graphene flakes at a relatively high yield (~ 1.3 mg min^−1^), was achieved in a single step by employing microwave plasma (see supplementary material) operating at atmospheric pressure conditions^40–44^. An ethanolic solution of ammonia (4 *wt* %) was used as precursor of both carbon and nitrogen. Argon was used as a background gas. The plasma properties have been tailored to achieve selective synthesis of free-standing N-graphene sheets. The flowing sheets were submitted to UV and IR irradiation in the post-plasma zone during their flight to the collecting system to further improve their structural quality^40^. Raman spectroscopy, SEM and TEM and XPS were used to characterize the morphology, microstructure and chemical properties of the synthesized material. The outlet gas stream was analyzed by Fourier-Transform Infrared spectroscopy (FT-IR) to detect the generated by-products. The electrical conductivity of the synthesized NG samples was determined using the Van der Pauw method^45^. Electrochemical measurements were also performed to elucidate the electrochemical behavior of NG for supercapacitor applications. Optical emission spectroscopy (OES) was used to detect the emission of plasma generated “building units” and to determine the gas temperature.

## Results

### High energy density plasma medium and outlet gas stream

**Figure 1:**
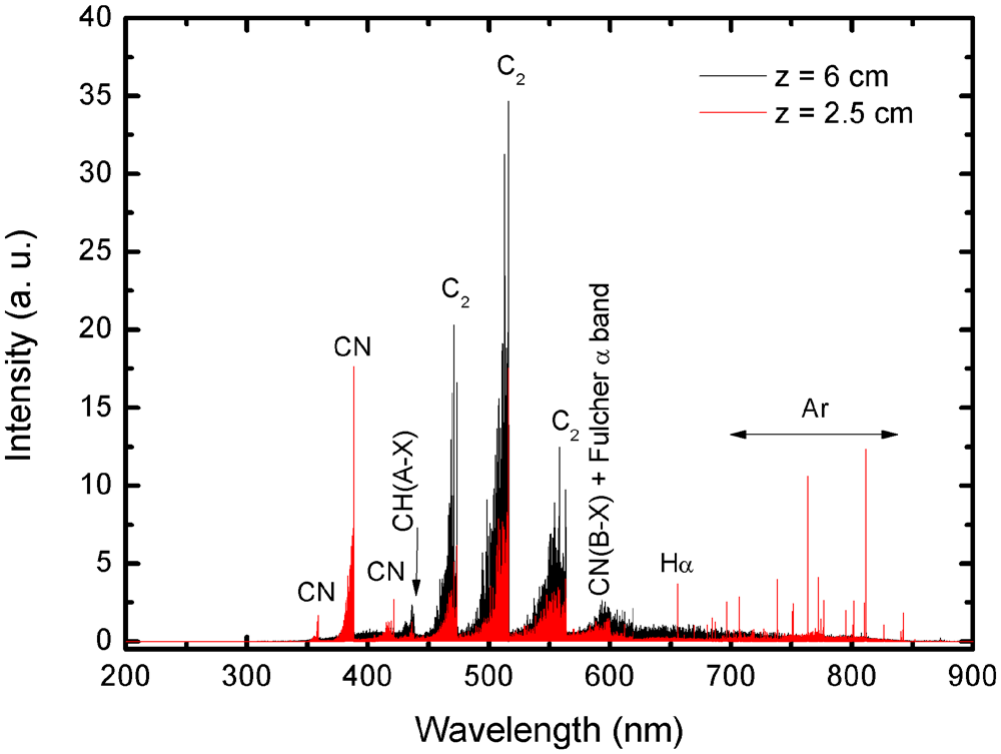
Plasma emission spectra at two axial positions along the discharge column (z refers to the distance from the launcher). (*Q*_*Ar*_ = 1200 sccm, *Q*_*Ar/Et/Am*_ = 100 sccm, P = 2 kW).

To identify the species of interest (C_2_, C, CN, N) and to determine important plasma parameters such as the gas temperature in the “hot” zone of the plasma reactor, emission spectroscopy has been applied. Optical emission spectroscopy allows the identification of the active carbon/nitrogen species in the plasma that are the “building units” for the synthesis and growth of the nanostructures in the mild plasma zone^39–43^. The detected argon/ethanol/ammonia plasma emission spectra in the visible range (240–750 nm) are shown in Fig. 1. The emission spectrum of argon/ethanol/ammonia plasma reveals the presence of new molecular and atomic species such as CN (violet system, B_2_Σ → X^2^Σ), C_2_ (Swan system, between 450–570 nm, A^3^Π_g_ → X^′3^Π_u_), CH band, the hydrogen Balmer-alpha line Hα (656.3 nm) and several Ar lines. These species are formed as a result of ethanol/ammonia decomposition and intensified chemistry in the “hot” plasma zone. As it can be seen in Fig. 1 strong plasma emissions of C_2_ molecular band, i.e. the Swan system, with heads band at 473.7, 516.5 and 558.6 nm were detected. The typical green colour of the plasma (see supplementary material) is due to C_2_ emission caused by the radiative decay of the excited state C_2_^*^(A^3^Π_g_). Electron impact and three body recombination processes, involving carbon and argon atoms, are responsible for the excitation of the ground state C_2_ molecules to the C_2_^*^(A^3^Π_g_) excited state, while CN species are formed according to the reaction C + N + Ar → CN*(B^2^Σ) + Ar^44^.

**Figure 2:**
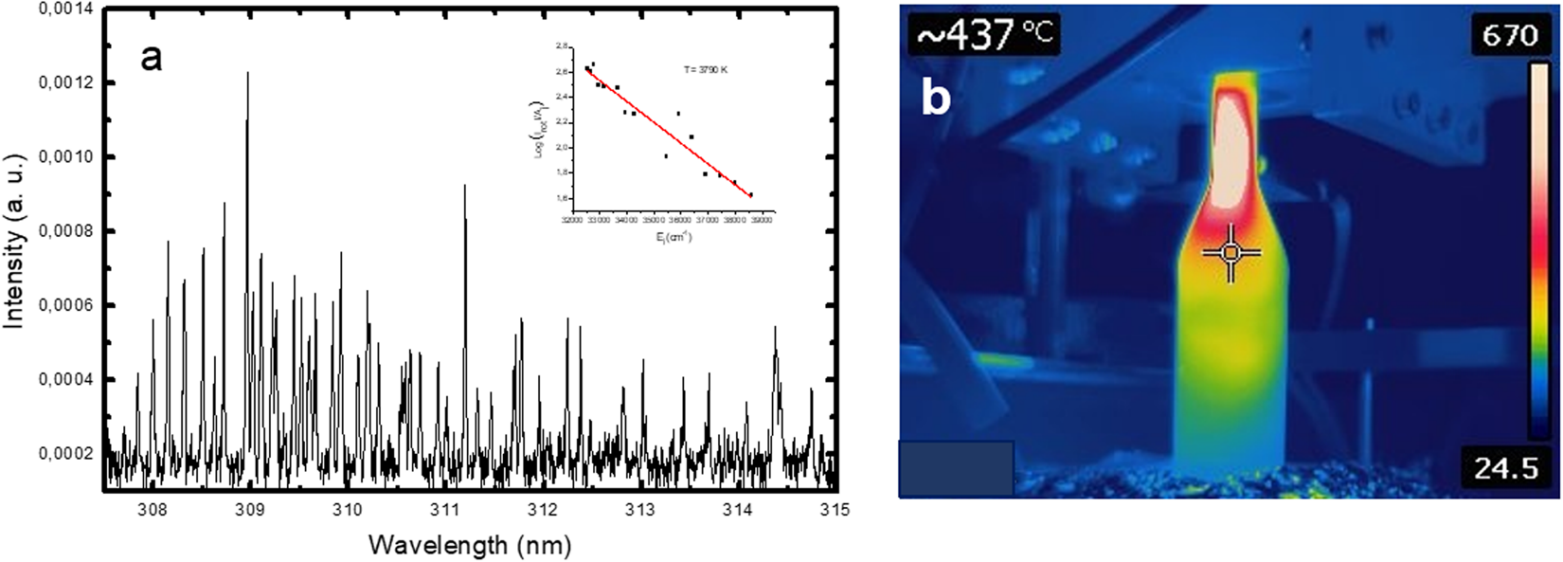
(**a**) Emission spectrum of the OH rotational bands; (**b**) 2D thermal map of the plasma reactor. (*Q*_*Ar*_ = 1200 sccm, *Q*_*Ar/Et/Am*_ = 100 sccm, P = 2 kW).

The gas temperature in the plasma, particularly in the “hot” zone, is an essential parameter regarding plasma kinetics. In gas discharges, the gas temperature can be usually assumed equal to the rotational temperature of diatomic molecular species^44^. At atmospheric pressure conditions the collision rates are high enough to provide local thermodynamic equilibrium. The rotational lines intensity of Q_1_ branch of the OH (A^2^Σ^+^, υ = 0 → X^2^Π_i_, υ´ = 0) (3000–3200 Å) band was used to estimate the rotational temperature, which at atmospheric pressure conditions is nearly equal to the gas temperature. The OH spectrum detected in the range 307.5–315 nm, (*Q*_*Ar*_ = 1200 sccm, *Q*_*Ar/Et/Am*_ = 100 sccm, P = 2 kW) at axial distance z = 2.5 cm from the launcher in the “hot” plasma domain is shown on Fig. 2(a). Using classical Boltzmann plot method (see the inset in Fig. 2(a)) the estimated rotational temperature is T ≈ 3800 K. It should be mentioned, that this temperature is associated with the axis of the “hot” plasma column, since the radiation collected originates mainly from this region. The gas temperature exhibits a sharp radial gradient. As shown in the previous studies, the plasma reactor´s wall temperature, as a boundary value of the gas temperature, is much lower^40–44^. An exemplification of the 2D map of the temperature as obtained by an infrared-sensitive thermal imager (FLIR camera) is shown in Fig. 2(b). As seen, the temperature demonstrates sharp decrease both in radial direction and in the expanding radius section reaching nearly room temperature at about 30 cm away from the launcher. “Hot” and “mild” plasma zones as described in^40^ can be clearly distinguished. The temperature corresponding to the position of the marker is 437 °C.

**Figure 3:**
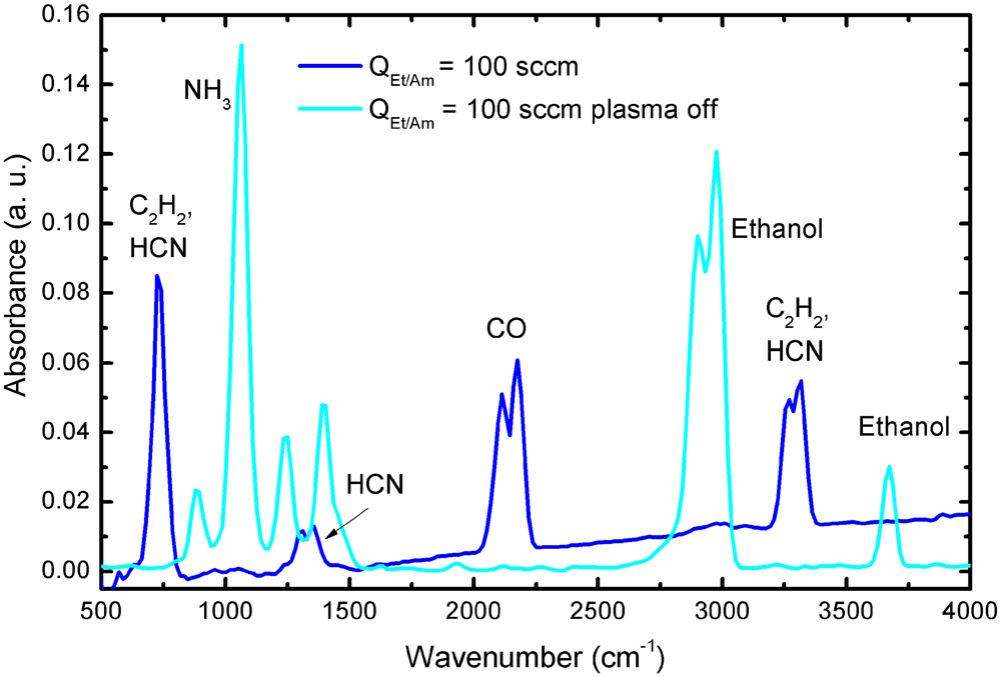
FT-IR spectra of the outlet gas stream with the plasma turned on and off (*Q*_*Ar*_ = 1200 sccm, *Q*_*Ar/Et/Am*_ = 100 sccm).

Additionally, FT-IR analysis of the outlet gas stream has been performed when the plasma is turned on or off (Fig. 3). The absorption spectra detected without plasma showed spectral lines at around 2900 and 3700 cm^−1^ and 1000 cm^−1^, which are “fingerprints” of the ethanol and the ammonia molecules, respectively. With the plasma turned on, these lines are not present, clearly indicating that the ethanol and ammonia molecules are decomposed in the plasma environment. At the same time, the absorption peaks of CO at around 2170 cm^−1^; C_2_H_2_ and/or HCN at around 720 and 3300 cm^−1^, and C_2_H_2_ at around 1350 cm^−1^ are detected (Fig. 3), demonstrating that the output gas flow contains some traces of CO, HCN and C_2_H_2_.

### Material characterization

**Figure 4:**
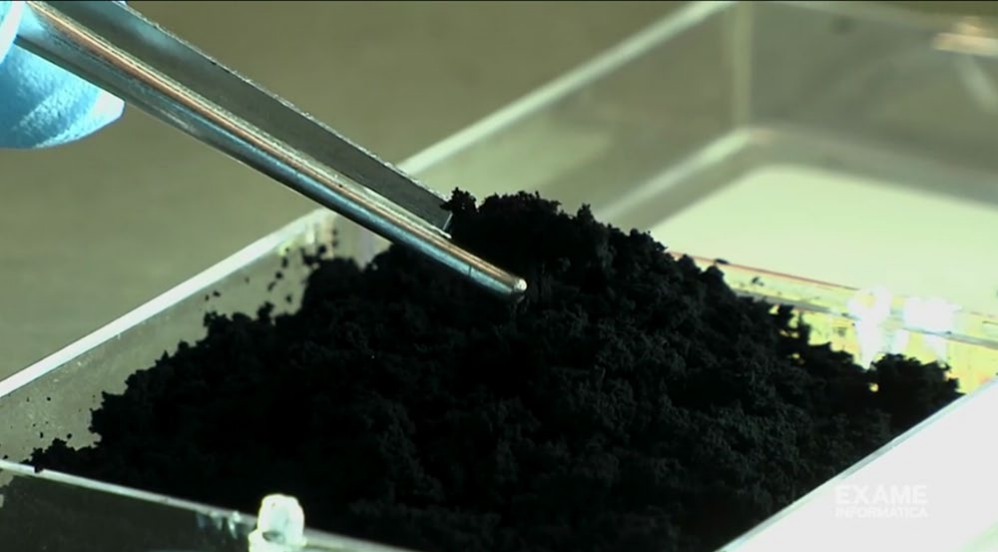
N-graphene sheets as synthesized.

**Figure 5:**
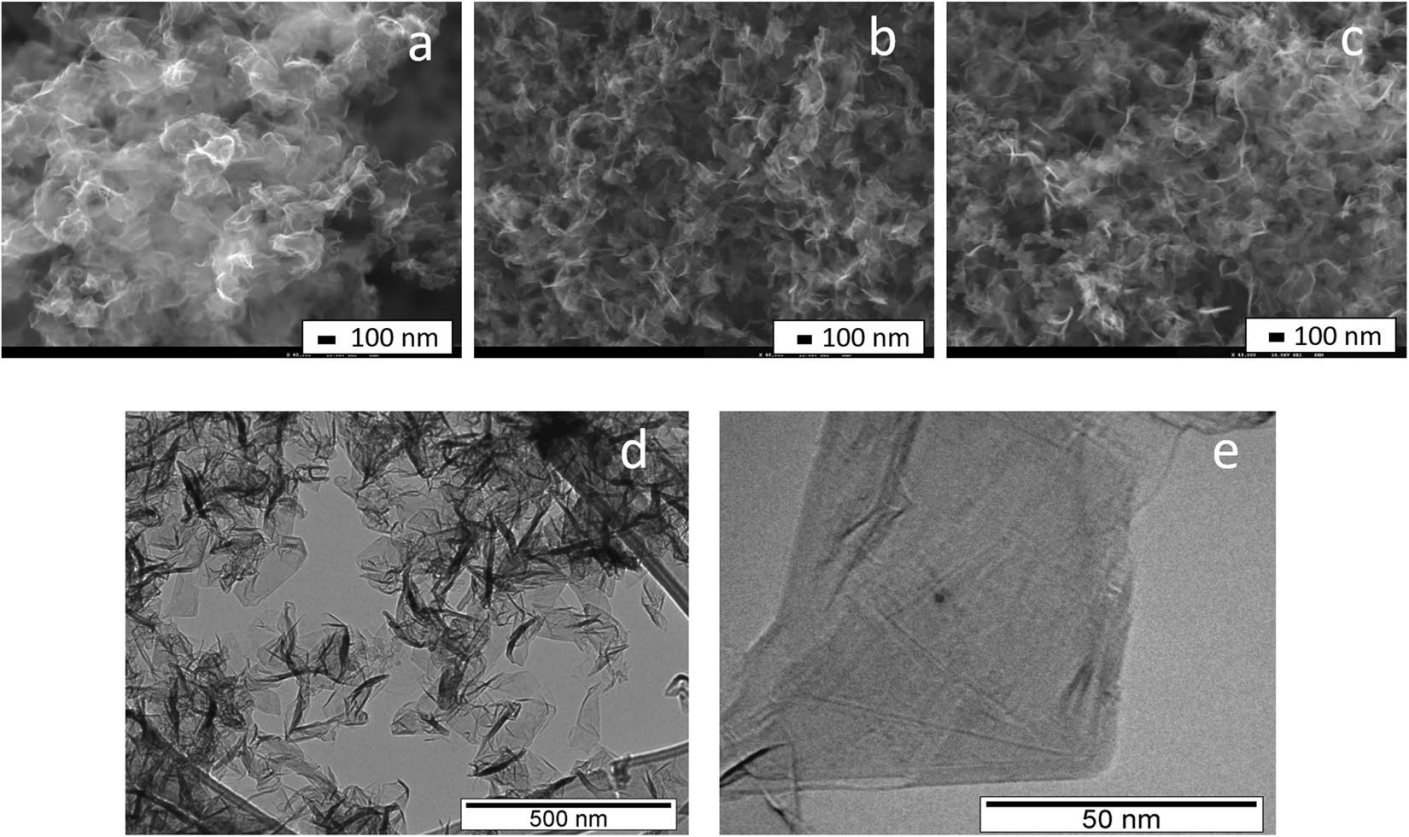
SEM images of N-graphene synthesized at P = 2 kW, *Q*_*Ar*_ = 1200 sccm, *Q*_*Ar/Et/Am*_ = 120 sccm: (**a**) as-obtained; (**b**) submitted to IR irradiation; (**c**) submitted to IR and UV irradiation; (**d**,**e**) TEM images of N-graphene obtained at P = 2 kW, *Q*_*Ar*_ = 1200 sccm, *Q*_*Ar/Et/Am*_ = 120 sccm and applied IR and UV irradiation.

The selective synthesis of N-graphene sheets at yield 1.3 mg.min^−1^ was obtained at background *Q*_*Ar*_ = 1200 sccm and precursor *Q*_*Ar/Et/Am*_ = 120 sccm gas flows and microwave power delivered to the plasma launcher P = 2 kW. Figure 4 shows ~0.5 g of the synthesized free-standing N-graphene sheets as a light fluffy powder. It is to be noted that the graphene sheets are stable at atmospheric conditions. Further engineering was applied by IR and UV irradiation of the produced sheets in the post-plasma zone. The value of the wall temperature was used as a measure of the IR irradiation. The UV irradiation of 4 W was applied to the sheets stored in a glass container (see supplementary material). Typical SEM images of the synthesized samples, presented in Fig. 5(a–c), reveals the inherent graphene morphology, i.e. paper-like and curved tiny structures. The morphology of the samples does not change noticeably after the IR and IR + UV irradiation.

TEM images of the synthesized N-graphene are shown in Fig. 5(d,e). As seen in these images, there are very thin sheets, as wide as several hundred nanometers. The darker areas observed correspond to regions with wrinkles, folding and overlapping of the sheets. The thin sheets with homogeneous and featureless appearance are likely to correspond to single or few layers graphene sheets.

Raman spectroscopy is an effective and conventional tool used to study the structural characteristics of graphene and its derivative N-graphene. Recent theoretical and experimental findings allowed the use of Raman spectroscopy to analyse and define many structural parameters, such as the size of graphene flakes and the number of graphene sheets in them, depending on the intensity, position and width of the Raman lines, identification of single versus multi-graphene layers, etc^46–51^.

**Figure 6:**
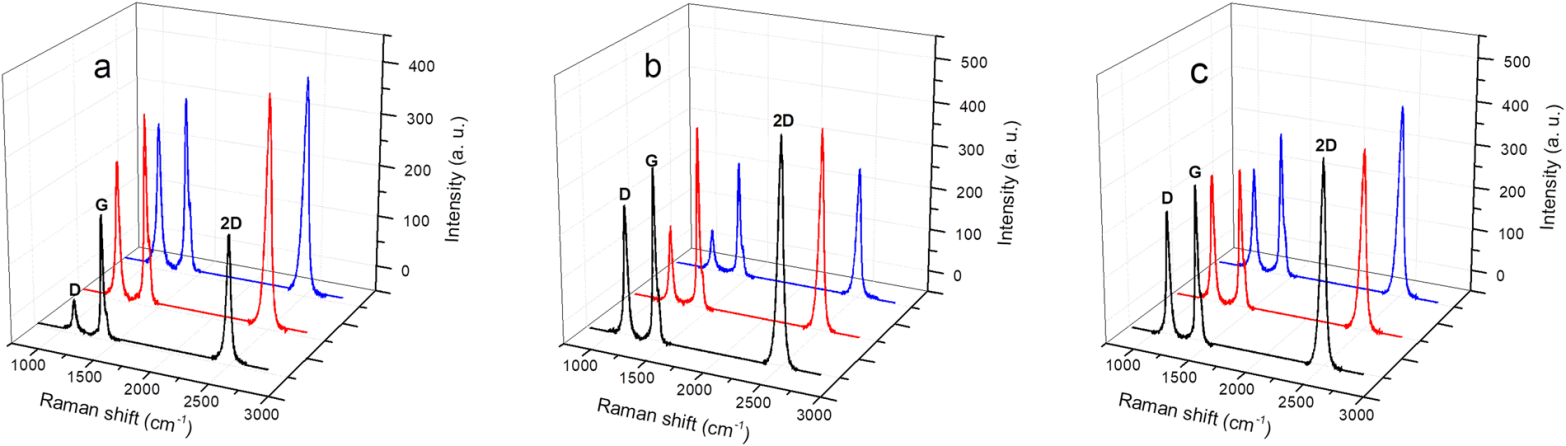
Raman spectra of N-graphene produced at P = 2 kW, *Q*_*Ar*_ = 1200 sccm, *Q*_*Ar/Et/Am*_ = 120 sccm: (**a**) as-obtained; (**b**) submitted to IR irradiation; (**c**) submitted to IR and UV irradiation.

The Raman spectra obtained from N-graphene samples synthesized in the conditions described in Fig. 5(a–c) are shown in Fig. 6(a–c). The spectra were collected from three different randomly chosen locations of the sample under investigation.

As seen from the figures the spectra are dominated by three peaks, attributed to the D, G and 2D bands at about ~1332 cm^−1^, 1583 cm^−1^, and 2658 cm^−1^, respectively. The G peak is a primary in-plane vibrational mode, common to all sp^2^ carbon systems. The D-band, commonly called ‘disorder‘ band, is due to the breathing modes of six-atom rings and requires a defect for its activation. The possible defects can be due to bond-angle disorder, bond-length disorder and hybridization, dangling bonds at the flake edges as well nitrogen atoms incorporated in graphene scaffold. The 2D-band is an overtone of the disorder-induced D-band and it is the most significant band in the Raman spectrum of graphene, thus confirming that the fabricated samples are really graphene. The full width at half maximum (FWHM) of the 2D peak in the synthesized samples is typically 50 cm^−1^, suggesting that graphene dominantly consists of several layers. For the sample obtained without application of IR and UV irradiations, the intensity ratio of the D-band to G-band at different locations on the sample vary significantly, as seen from Fig. 6(a), from 0.26 to 0.83, while the intensity ratio of the 2D-band to G-band varies from 1 to 1.2. Similar behavior is observed in Fig. 6(b) for the post-plasma IR irradiated material. The results demonstrate a non-homogeneous defects distribution on the graphene scaffold. However, by applying simultaneously IR and UV irradiation an increase of the intensity ratio of the D-band to G-band up to ~0.9 is observed. This ratio is nearly constant for the three different locations on the NG sample Fig. 6(c). This gives some confidence to the hypothesis that a more homogeneous distribution of the defects, in particular N-doping, of graphene scaffold is achieved. Besides, the possible reason for the increase of the intensity of the D-line may be the increase of the N doped sites.

*Ex-situ* XPS analysis has been performed to study the relative extension of the delocalized system, the amount and type of nitrogen atoms doping the graphene and the amount of oxygen.

**Figure 7:**
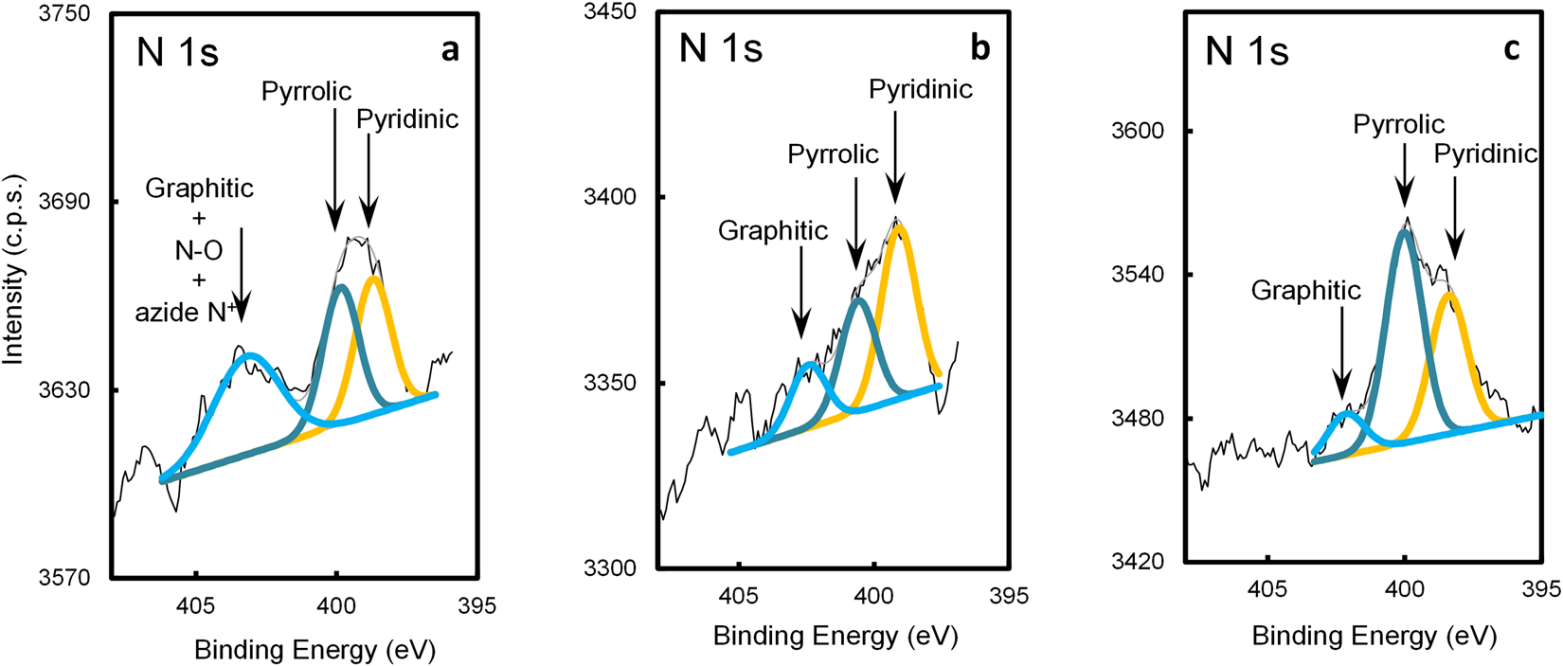
Detailed XPS N 1s regions of N-graphene samples produced at P = 2 kW, *Q*_*Ar*_ = 1200 sccm, *Q*_*Ar/Et/Am*_ = 120 sccm: (**a**) as-obtained; (**b**) submitted to IR irradiation; (**c**) submitted to IR and UV irradiation. Spectra were smoothed with moving averages (width = 0.8 eV).

Nitrogen was detected in all the samples. The detailed XPS regions (Fig. 7) show different N 1s components corresponding to nitrogen atoms in different positions in the graphene lattice. N 1s was fitted to 3 contributions centered at 398.7 ± 0.3 eV, 400.2 ± 0.3 and 402.3 ± 0.1 eV attributed, respectively, to pyridinic, pyrrolic and graphitic nitrogen^40^. However, N 1s spectrum shown in Fig. 7(a), presents a slightly wider peak than those fitted in the other samples, centered at 403.2 eV, which, besides graphitic nitrogen, can also include electron-deficient nitrogen atoms from any azide groups^52^ and/or nitrogen atoms bound to oxygen, like nitroso groups.

**Figure 8:**
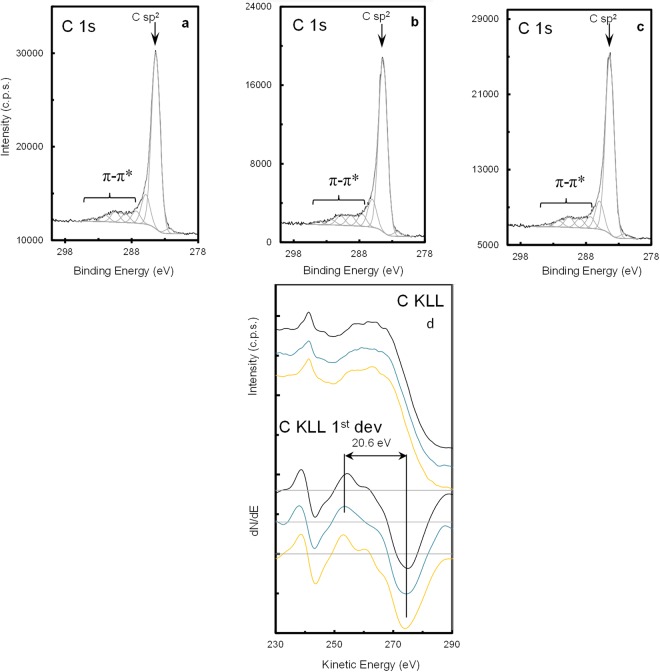
XPS C 1s regions and Auger C KLL structures and respective 1^st^ derivatives of N-graphene samples synthesized at P = 2 kW, *Q*_*Ar*_ = 1200 sccm, *Q*_*Ar/Et/Am*_ = 120 sccm: (**a**) as-obtained; (**b**) submitted to IR irradiation; (**c**) submitted to IR and UV irradiation. (d) In C KLL and dN/dE the conditions correspond, from bottom to top, to those in (**a**,**b**,**c**), respectively. C KLL curves were smoothed with 11 successive moving averages (width = 1.8 eV).

C 1s regions, which are very much alike in all the samples (Fig. 8), show a main peak positioned at 284.4 ± 0.1 eV, assigned to sp^2^ carbon atoms in highly delocalized C-C bonds, and the expected features due to the energy losses associated to π-π* excitations. This energy losses region can extend roughly from 287 eV to over 295 eV, superposing to much less intense photoelectron peaks assigned to carbon bound to oxygen (in different chemical environments: epoxide, carbonyl, carboxylate), which, as attested by O 1s region (not shown), is in a residual amount (Table 1). From 285 eV to ~287 eV, sp^3^ carbon atoms singly bound to carbon, hydrogen, nitrogen and oxygen can be found added to a vibrational fine structure that broadens the main peak to the high binding energy side^53^. Also, the contribution of some energy losses, in this energy range, to π-π* excitations cannot be discarded given the high extension of π delocalization^53^. The peak centered at 282.3 eV is tentatively assigned to an energy gain due to the presence of holes in the π band.

**Table 1:** Atomic concentrations (%) and atomic ratios.

Sample		a	b (IR)	c (IR + UV)
**At. Conc. (%)**				
C		98.0	97.7	98.1
O		1.8	2.1	1.5
N	Pyridinic	0.10	0.11	0.14
	Pyrrolic	0.10	0.07	0.21
	Graphitic	0.13	0.04	0.04
**Atomic ratios**				
**N/C**		0.0033	0.0023	**0.0039**
**O/C**		0.018	0.021	**0.015**
**N/O**		0.19	0.11	**0.25**
**sp** ^**2**^ **%**		69.9	70.4	**70.9**

Also, C KLL Auger regions and, in particular, their first derivatives can confirm the strong electron delocalization of these carbon structures (Fig. 8): the D parameter, defined as the distance (energy difference) between two consecutive absolute maximum and minimum, is equal to 20.6 eV for the sample synthesized under simultaneous IR and UV irradiation. This value is close to the one reported for graphite^53^. All samples have similar D parameter, but since some uncertainty is introduced when computing this parameter from C KLL data arbitrarily smoothed, no further conclusions can be drawn from these parameters. Nevertheless, the differences detected in the fine dN/dE Auger structure, must be related to the slightly different relative amounts of nitrogen detected: samples **a** and **c**, that have more nitrogen and are less oxidized than sample **b**, show a relative maximum at ~260 eV in the Auger fine structure of dN/dE (Fig. 8) which is absent in sample **b**. This small difference can be related to different local density of the valence states which depend on the chemisorbed species. Table 1 shows some relevant quantitative results.

The results in Table 1 demonstrate that the sample submitted to additional IR and UV irradiation presents the largest sp^2^% (=C_sp_^2^/C_Total area_ × 100) and also has the largest relative amount of nitrogen (N/C ~ 0.4%) and is the least oxidized (O/C = 1.5%). The sequence of UV irradiation and relatively low heating (i.e. 200 °C) slightly increases the sp^2^%. Additionally, changes in the relative amount of the different nitrogen groups are observed, such as an increase of the amounts of pyridinic and pyrrolic nitrogen. This might be caused by activation of the defects due to additional heating, with the subsequent bonds reorganization. The fact that these changes are small, however, makes it difficult to assess the validity of this assumption and further investigation to analyze this influence is an ongoing work. The small decrease of oxygen/carbon ratio from 0.021 to 0.015 can be attributed to the kicking-out of the oxygen groups by UV irradiation with a photon energy of ~3 eV.

The electrical conductivity of the N-graphene sheets was measured on pressed disc pellets of 8 mm diameter and thickness between 1.2 and 3.2 mm. A four-contact configuration with pinching point contacts located on the periphery of the pellet, was used, providing longitudinal conductivity measurements for the current lines parallel to the pellet surface. For the current ranging from 0.5 to 3.5 mA, the current-voltage characteristics and reconfirmation of the Ohmic contact character were obtained.

The values of electrical conductivity presented in Table 2 are averaged over several measurements. As can be seen (Table 2) the electrical conductivity depends on the pellet’s density. However, some correlation between measured conductivity and the percentage of pyridinic and pyrrolic nitrogen groups can be noted. The increase of the percentage of pyridinic and pyrrolic as well as oxygen groups results in a decrease of conductivity. It should be noted, that the electrical conductivity values measured in the present study present much higher values than the one obtained for graphene produced by chemical methods, clearly demonstrating the benefit of the plasma synthesis method used in the present study^54^.

**Table 2:** Electrical characteristics.

Samples	Density (g/cm^3^)	ρ (Ω.cm)	Conductivity (S.m^−1^)
(a)	1.25	0.032	3125
(b)	1.14	0.06	1666
(c)	1.2	0.04	2500

### Electrochemical results

N-graphene, produced at P = 2 kW, *Q*_*Ar*_ = 1200 sccm, *Q*_*Ar/Et/Am*_ = 120 sccm and submitted to IR and UV irradiation, was tested as electrode for supercapacitor applications by performing cyclic voltammetry (CV), galvanostatic charge-discharge (GCD) and Electrochemical Impedance Spectroscopy (EIS) in 1 M H_2_SO_4_ electrolyte. The measurements were performed in a three-electrode electrochemical cell using Pt and Standard Calomel Electrode (SCE) as counter and reference electrodes, respectively.

**Figure 9:**
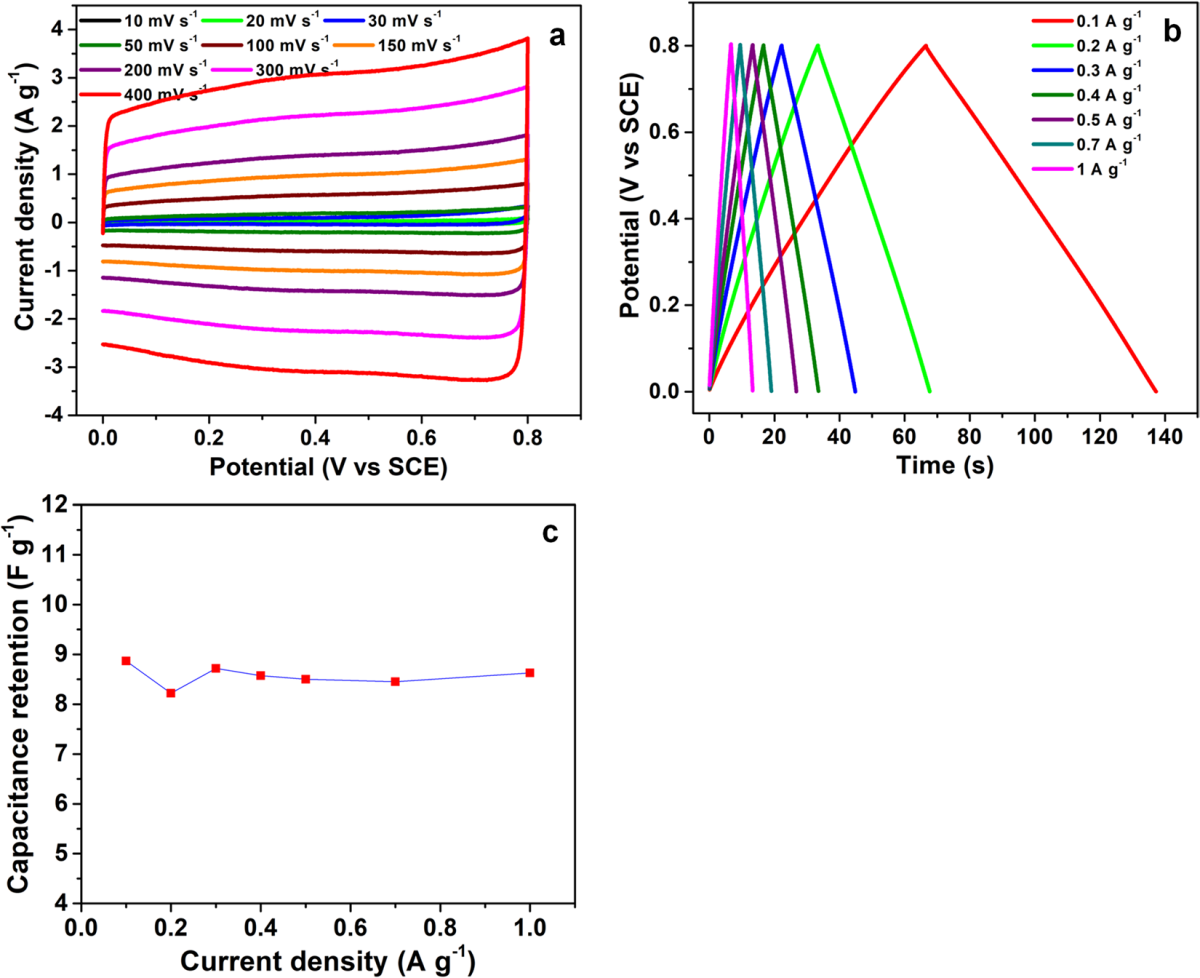
(**a**) Cyclic voltammetry of N-graphene at different scan rate; (**b**) galvanostatic charge discharge measurement at different current density; (**c**) specific capacitance *vs*. current density.

The CV curves obtained in the potential window of 0–0.8 V *vs*. SCE at varying scan rates, are presented in Fig. 9(a) and evidence an almost perfect rectangular behavior, as expected for a material storing charge thanks to an electric double layer (EDLC) mechanism. The nearly rectangular voltammogram also indicates the absence of resistive features, meaning that N- graphene possesses very high electronic conductivity. The consecutive increase in the anodic and cathodic current response on increasing the scan rate accounts for the occurrence of highly reversible electrochemical processes at the electrode/electrolyte interface and this behavior was perfectly retained even at high scan rates, in this case 400 mVs^−1^. Such rectangular features and high reversibility can be attributed to the high and easily accessible active surface area of the N-graphene.

GCD measurements were also performed by applying different current densities ranging from 0.1 to 1 Ag^−1^ (Fig. 9(b)). A linear charge and discharge behaviour at all current densities confirms the EDLC behavior of charge storage. It is worth to mention that the curves do not show any internal resistance (IR) drop and evidence coulombic efficiency (ratio of discharge to charge time) of 100%, meaning that N-graphene possesses very good electronic conducting properties. The gravimetric capacitance and the rate capability was calculated using the equation below:

$${C}_{s}=(I{\rm{\Delta }}t)/{\rm{\Delta }}V$$

where, *I* is the current density (Ag^−1^), ∆t is discharge time (s), and ∆V is working potential window. The specific capacitance of 8.87 Fg^−1^ was obtained at current density of 0.1 Ag^−1^ which retained ~100% of the capacitance on increasing the applied current ten times higher i.e. at 1 Ag^−1^ (Fig. 9(c)) showing the excellent rate capability of the material. Despite the fact of having low specific capacitance such materials can be used to formulate composites to enhance the electronic conductivity of redox based metal compounds, which generally possess high resistive nature and require improvement in the overall performance for supercapacitor/energy storage devices^12^. The specific capacitance of this material can be further increased by optimizing pore size distribution and by increasing the nitrogen doping level, which is ongoing work and follow up in next publications.

**Figure 10:**
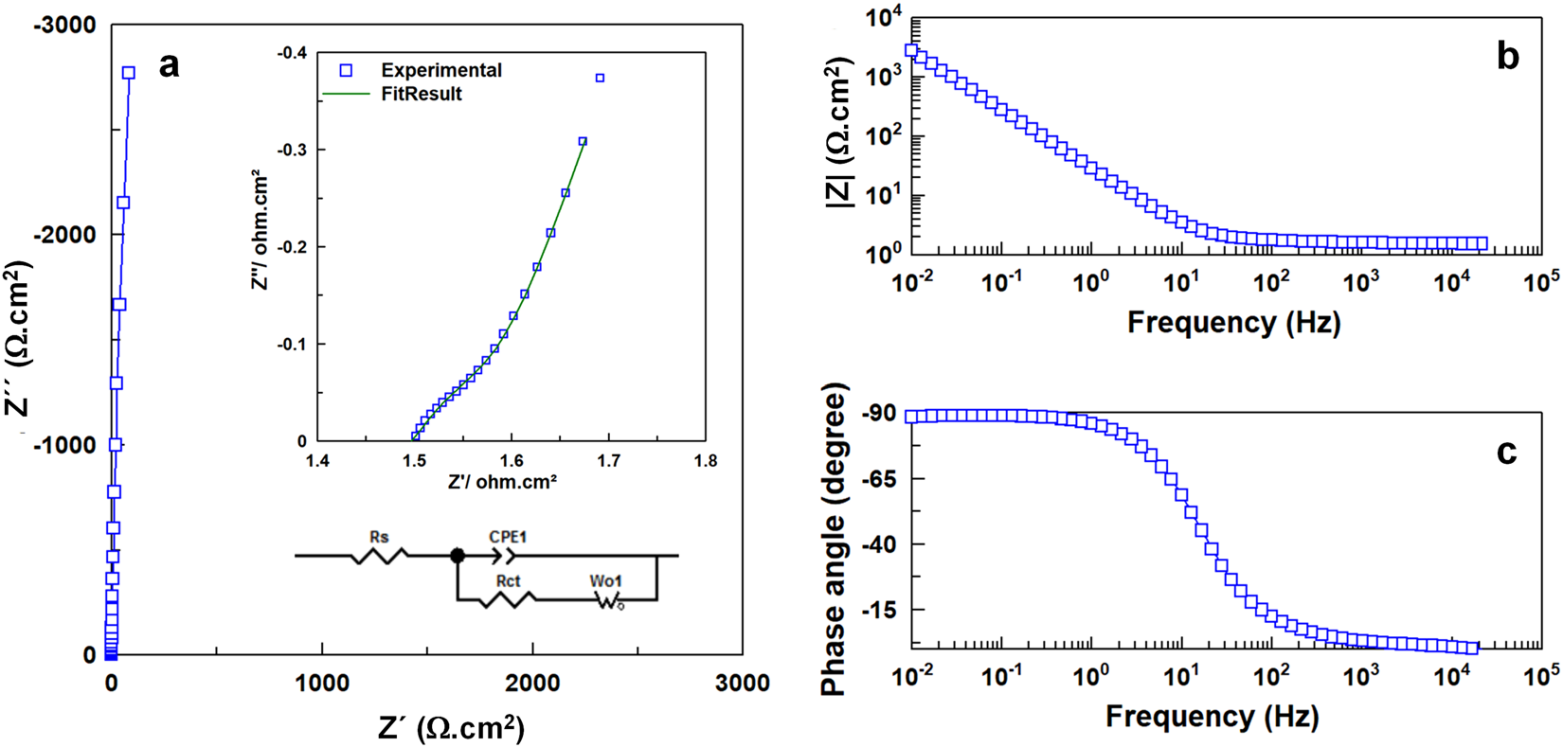
Electrochemical impedance spectroscopy (EIS) plots: (**a**) Nyquist plot (inset showing the magnified image and equivalent circuit diagram, green line is the fitted curve of experimental data), (**b**,**c**) Bode plots.

EIS was performed at open circuit potential (OCP) to study the resistive nature of the synthesized N-graphene. The spectra along with the equivalent circuit proposed to fit the data are illustrated in Fig. 10 and the fitted parameters are given in Table 3.

**Table 3:** Electrochemical impedance parameters obtained after fitting the experimental data.

Fitting Parameters		Value
R_s_ (Ωcm^2^)		1.49
R_ct_ (Ωcm^2^)		0.045
CPE (n)		0.98
Warburg element, W_o_	(Ωcm^2^)	0.386
	n	0.51
χ^2^ value		3.08 × 10^−5^

Nyquist plot gives the information about the charge transfer resistance at high frequency and capacitive at low frequency. The absence of a semi-circle in the high frequency region, as can be noticed from magnified plot shown in inset of Fig. 10(a), and a very low value 0.045 Ωcm^2^ obtained from fitting suggest negligible charge transfer resistance.

The emergence of Warburg behaviour denoted by W_o_ in the mid frequency region represents the diffusion of ions dependent on frequency. A well-defined and short Warburg region portion, with phase angle of around - 45° (n = 0.51), evidences the short and equal diffusion path length of the ions in the electrolyte. Also, the Warburg resistance value of 0.386 Ωcm^2^ was very low. The very low charge transfer resistance and the decreased diffusion effect can be the reason for very fast reversible processes at the interface, which explains the rectangular CV, the rectangular nature retention even at high scan rates and the linear profiles of GCD plots. At very low frequency, the presence of almost vertical line and phase angle close to - 90° (can be noticed from Bode plot - Fig. 10(b)) with n value of 0.99 indicates an almost ideal supercapacitor response.

## Conclusion

Microwave plasma-based method for direct synthesis of free-standing N-graphene sheets was applied at atmospheric pressure conditions. Nitrogen doping of 0.4% has been obtained at a single step using ethanol and ammonia as carbon and nitrogen precursors. The method is fast, highly cost-efficient, and does not depend on catalyst, vacuum systems and multistep cumbersome procedures. Applying infrared (IR) and ultraviolet (UV) irradiation to the flow of free-standing sheets in the post-plasma zone carries out changes in the percentage of sp^2^%, the N doping type and the oxygen functionalities. A detailed study on the nature of these changes is under way. The main advantage of the approach here presented is the control over the energy and material fluxes towards growing nanostructures via proper reactor design and tailoring of the plasma environment in a synergistic way.

Electrodes assembled with the produced N-graphene showed good electrochemical response with negligible interfacial resistance, even at low percentage of N-doping in the graphene, evidencing high potential for supercapacitor applications.

By optimizing the microwave plasma reactor performance, along with detailed modelling of the plasma environment and formation of the main “building units”, well controlled synthesis of carbon/nitrogen atom lattices can be generated. Further investigations are underway in these directions, including the use of other carbon/nitrogen precursors such as methane, air, pyridine etc., to achieve further advancements in this technique, and increase the yield along with reduction of the cost via scaling-up the plasma reactor. Focus will be on mastering the process by achieving control on both the level of doping and bond configuration.

The method is highly scalable and versatile: carbon precursors in liquid, gas and solid state can be used and different graphene derivatives can be synthesized using the same plasma reactor. Moreover, customization is possible, i.e., depending on the target application one can tailor N-graphene sheets in a suitable form and required quality.

## Electronic supplementary material

Supplementary Material
