# Supplementary material for: Large-scale synthesis of free-standing N-doped graphene using microwave plasma
Source: Sci Rep. 2018 Aug 22;8:12595. doi: 10.1038/s41598-018-30870-3 (PMC6105711; doi:10.1038/s41598-018-30870-3)
Supplement: Supplementary file 1 — Supplementary Material [file 41598_2018_30870_MOESM1_ESM.docx]

**Supplementary Material**

**Large-scale synthesis of free-standing N-doped graphene using microwave plasma**

N. Bundaleska^1^, J. Henriques^1^, M. Abrashev^2^, A.M. Botelho do Rego^3^, A.M. Ferraria^3^, A. Almeida^4^, F.M. Dias^1^, E. Valcheva^2^, B. Arnaudov^2^, K.K. Upadhyay^5^, M.F. Montemor^5^, E. Tatarova^1*^

**Experimental setup and synthesis method**

A surfatron-based setup was used to create a surface wave induced microwave plasma at atmospheric pressure conditions as shown in Fig. 1 [1]. The microwave power is provided by a 2.45 GHz generator (Sairem), whose output power was set to 2000 W. The generator is connected to a waveguide (WR-340) system, which includes an isolator, directional couplers, a 3-stub tuner and a waveguide-surfatron as the field applicator. The system is terminated by a movable short-circuit. The discharge takes place inside a quartz tube reactor, which is inserted downstream vertically and perpendicularly to the waveguide wider wall. The quartz reactor comprises two sections; a small one with internal and external radii of 0.75 cm and 0.9 cm, respectively, connected via conical section to the large tube with internal and external radii of 2.15 cm and 2.3 cm. A second quartz tube is used to introduce the vaporized precursor, *i.e.*, ethanol + ammonia molecules, in the discharge zone. The background argon gas is injected into the discharge tube at flow rates (*Q_Ar_*) varying from 1000 to 1500 sccm under laminar gas flow conditions. Part of the background Ar gas flow passes through a tank filled with the precursor, placed inside of sonication bath, with accurate control of the temperature to drag the ethanol/ammonia (4 *wt* %) molecules. The precursor partial flux (*Q_Ar/Et/Am_*) was varied in the range 50 to 120 sccm. The total gas flow is *Q^tot^ = Q_Ar_*+ *Q_Ar/Et/Am._*. Gas flow rates are controlled by a MKS247 Readout coupled to two MKS flow meters. To increase control over the synthesis process and improve the structural quality of the assembled flowing nanostructures both infrared (IR) and UV irradiation were applied in the gas-phase zone, where assembled nano-structures are dragged by the axial gas flow. The system with a network of IR lamps (length 20 cm) is placed immediately after the end of the plasma zone (in the range 15-30 cm away from the launcher). The 2D map of the temperature, as a measure of applied heating (IR irradiation) was monitored with FLIR thermal imager. The nanostructures were captured by a tornado type cyclone system followed by a water trap to capture the nanostructures that are able to escape with the gas flow. The N-graphene powder collected in a glass container was further irradiated with UV lamps emitting in the range 300-400 nm at about 4 W total UV power to further modify their structural properties.


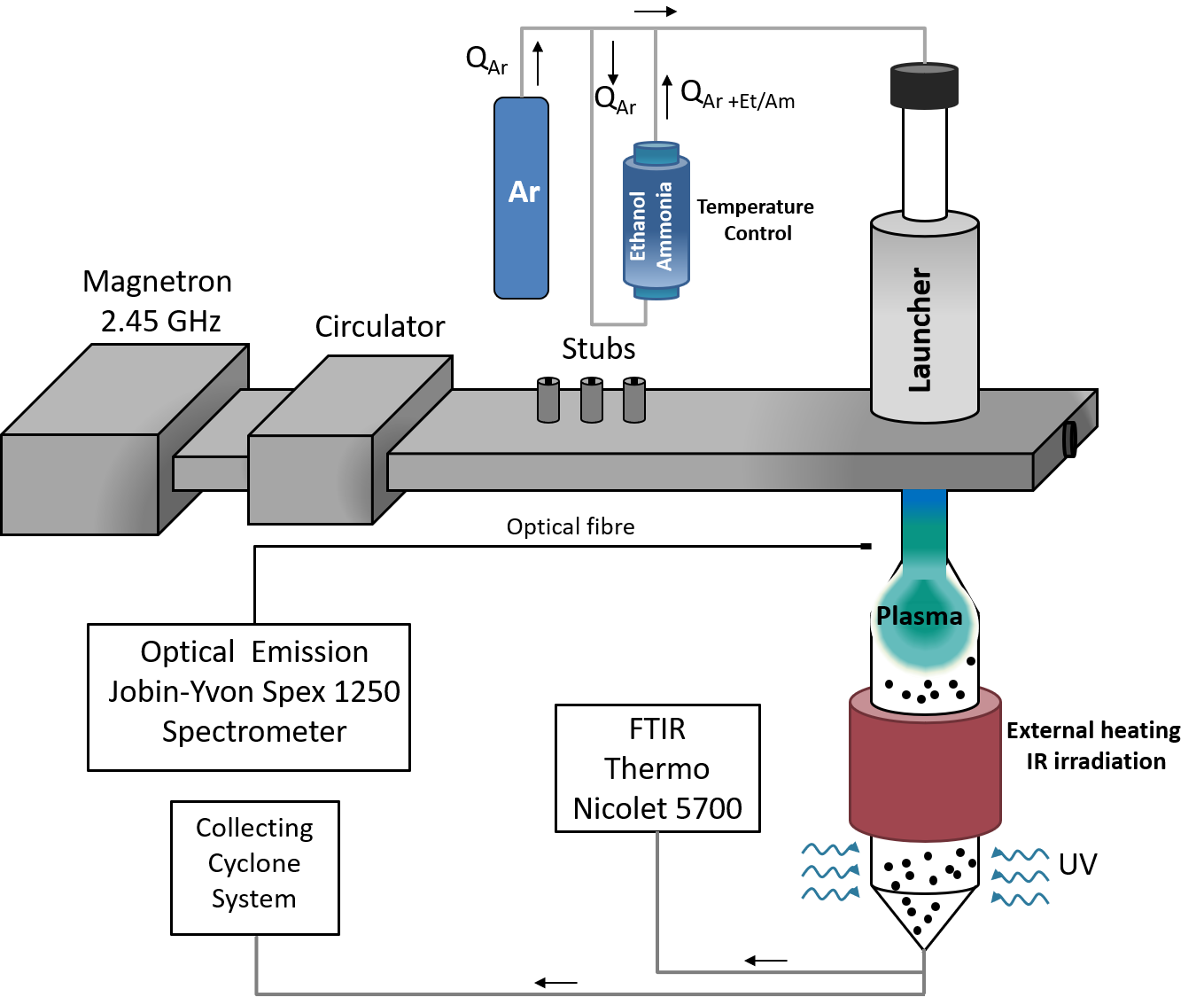


Figure 1. Experimental setup

For gas sampling, a portion of the output gas stream from the plasma reactor (close to the late plasma afterglow region) was directed to a FT-IR Thermo Nicolet 5700 spectrometer and the absorption spectra of species was detected in the wave number range 1000 – 4000 cm^-1^. To monitor in real time the main "building units", i.e., carbon and nitrogen species, the light emitted by the plasma was collected perpendicularly to the discharge tube by an imaging optical fiber and directed to the entrance of a Jobin-Yvon Spex 1250 spectrometer (1200/2400 g/mm grating) equipped with a CCD camera. The cryogenic, back illuminated UV sensitive CCD camera has a 2048×512 matrix, featuring a 13.5 µm pixel-size, which provides high spectral resolution. A collimator located in front of the optical fiber defines the discharge volume (at the discharge axis) from where the plasma radiation is collected. The plasma emission spectra in the 230−750 nm range have been investigated.

SEM characterization of the samples has been performed using a JEOL, JSM-7001F field emission gun scanning electron microscope operating in secondary electron imaging mode (SEI) using 15kV accelerating voltage. The samples were deposited on a double-sided carbon tape mounted on an aluminium stub.

To probe the phonon structure of the nanostructures, and to provide a quick and easy structural and qualitative characterization of the samples Raman spectroscopy analysis was performed. The synthesized nanostructures were freely suspended on a glass substrate and the Raman spectra from different regions on the substrate were obtained using a LabRAM HR Visible (Horiba Jobin-Yvon) Raman spectrometer with 1 cm^-1^ spectral resolution and 633 nm He-Ne laser excitation with laser spot size of 2 μm. Measurements were performed with a laser power *P_l_* = 0.054 mW to avoid overheating.

Free-standing N-doped graphene sheets were characterized by X-ray photoelectron spectroscopy using a XSAM800 spectrometer from KRATOS with an incident X-radiation from a Mg Kα source (1253.6 eV). Operating conditions and spectra acquisition parameters are detailed elsewhere [2]. Fluffy powder samples were mounted on the XPS holder with a double face tape. No silicon was detected discarding any interference of the tape spectrum. No charge correction was needed, since the C 1s main peak, detected at 284.4 ± 0.1 eV, is typical of aromatic C-C or C-H in graphene [3]. The sensitivity factors (from Vision 2 library) used for quantification purposes were 0.318 for C 1s, 0.736 for O 1s and 0.505 for N 1s.

The graphene flakes were placed directly as a solid powder onto transmission electron microscopy (TEM) copper grid. The sheets were then characterized by a Hitachi H8100 TEM, operating at 200 kV accelerating voltage.

To measure the electrical conductivity of the graphene sheets, N-graphene powder was pressed into discs of 8 mm diameter and 1.2 mm thickness. The electrical conductivity was measured applying the Van der Pauw method at room temperature [4].

**References**

# [1] Moisan, M., Zakrzewski, Z. Plasma sources based on the propagation of electromagnetic surface waves. *J Phys D: Appl. Phys.* 24 1025 (1991).

[2] Carapeto, A. P., Ferraria, A. M., Botelho do Rego, A. M. Unraveling the reaction mechanism of silver ions reduction by chitosan from so far neglected spectroscopic features. *Carbohydrate Polymers* **174**, 601–609 (2017).

[3] Tatarova, E., et al. Towards large-scale in free-standing graphene/N-graphene sheets”, *Scientific Reports* **7**, Article number: 10175 (2017), doi:10.1038/s41598-017-10810-3.

[4] [Sze, S. M.](https://en.wikipedia.org/wiki/Simon_Sze), Lee, M.-K. Semiconductor Devices: Physics and Technology. New York: Wiley (2016).
